# On the Security of a PUF-Based Authentication and Key Exchange Protocol for IoT Devices

**DOI:** 10.3390/s23146559

**Published:** 2023-07-20

**Authors:** Da-Zhi Sun, Yi-Na Gao, Yangguang Tian

**Affiliations:** 1 Tianjin Key Laboratory of Advanced Networking (TANK), College of Intelligence and Computing, Tianjin University, Tianjin 300350, China; gaoyina@tju.edu.cn; 2 Department of Computer Science, University of Surrey, Surrey GU2 7XH, UK; yangguang.tian@surrey.ac.uk

**Keywords:** physically unclonable function, authentication, key exchange, insider attack, surveillance, impersonation

## Abstract

Recently, Roy et al. proposed a physically unclonable function (PUF)-based authentication and key exchange protocol for Internet of Things (IoT) devices. The PUF protocol is efficient, because it integrates both the Node-to-Node (N2N) authentication and the Node-to-Server (N2S) authentication into a standalone protocol. In this paper, we therefore examine the security of the PUF protocol under the assumption of an insider attack. Our cryptanalysis findings are the following. (1) A legitimate but malicious IoT node can monitor the secure communication among the server and any other IoT nodes in both N2N authentication and N2S authentication. (2) A legitimate but malicious IoT node is able to impersonate a target IoT node to cheat the server and any other IoT nodes in N2N authentication and the server in N2S authentication, respectively. (3) A legitimate but malicious IoT node can masquerade as the server to cheat any other target IoT nodes in both N2N authentication and N2S authentication. To the best of our knowledge, our work gives the first non-trivial concrete security analysis for the PUF protocol. In addition, we employ the automatic verification tool of security protocols, i.e., Scyther, to confirm the weaknesses found in the PUF protocol. We finally consider how to prevent weaknesses in the PUF protocol.

## 1. Introduction

**Figure 1:**
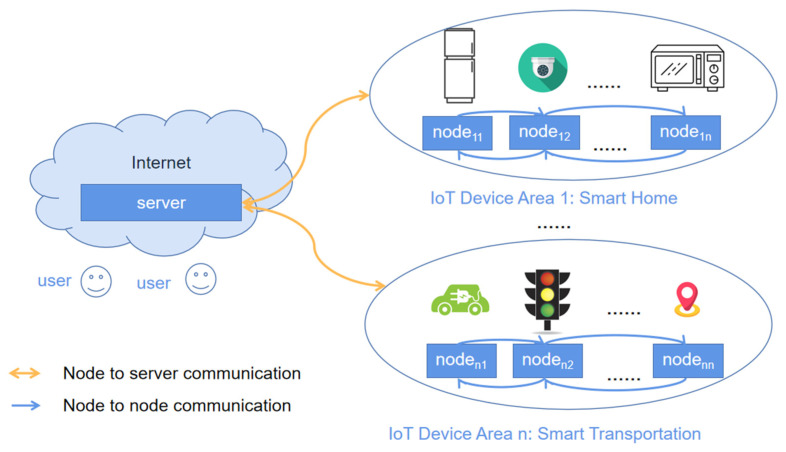
Architecture of IoT authentication network.

In recent years, the Internet of Things (IoT) has been growing rapidly and all kinds of IoT devices are often present in our personal lives in the form of various applications, such as smart homes, smart transportation, and smart cities. Figure 1 depicts the communication scenario between the IoT server and devices (nodes). However, security is one of the major challenges when IoT enters sensitive fields, e.g., a smart street light monitoring system. Unlike traditional networks, IoT consists of a large number of complex, heterogeneous, and interoperable IoT devices [1]. Hence, under a hostile environment, IoT devices are often vulnerable to attacks and therefore require stronger security techniques to overcome their potential security weaknesses, such as counterfeit identity and sensitive data leakage. Authentication and key exchange protocols can provide the service of authentication and secret session key establishment between various IoT devices. Here, the session key is used to support confidential data transfer between IoT devices.

However, designing authentication and key exchange protocols is an intractable task for IoT applications. Firstly, there is a huge number of IoT devices, but their computing and storage resources are usually limited. Therefore, lightweight protocols are urgently needed to realize efficient interaction between devices without overloading their performance. Secondly, IoT devices are often deployed in complex, heterogeneous network environments and can be connected to and accessed by multiple unknown and untrusted devices, leading to extreme security challenges. Robust authentication and key exchange protocols should satisfy all strict requirements from IoT devices and their applications.

The physically unclonable function (PUF) [2] can help build robust security protocols. PUF is regarded as a basic security primitive for resource-constrained IoT devices. PUF generates a unique digital fingerprint from micro-variations in physical devices. This fingerprint is unable to be cloned and is sensitive to being tampered with. PUF generates an unpredictable output (i.e., response) for corresponding input (i.e., challenge). The process is irreversible and is independent of storage memory. PUF is realized in various scenarios, such as field programmable gate array (FPGA) [3], cloud storage, and IoT, to protect data from unauthorized access and attacks. The advantage of PUF is that resource-constrained devices do not require the maintenance of the secret key [4]. Moreover, it reduces the risks associated with key transmission, storage, and management to a greater extent than other common cryptographic techniques. Therefore, PUF is as a promising alternative to designing lower-cost authentication and key exchange protocols.

On the one hand, PUF has advantages in efficiency and security compared with cryptographic algorithms. Hence, it is expected to design more practical authentication and key exchange protocols for IoT. On the other hand, PUF-based authentication and key exchange protocols are relatively new, compared with the protocols using purely cryptographic algorithms and/or smart cards. And there are still many security issues in its designs, which should be addressed. Hence, in this paper, we investigate the PUF-based authentication and key exchange protocol for IoT devices.

### 1.1. Related Work

Cryptographic algorithms are widely used in security protocols. According to the type of cryptographic algorithm, we divide PUF-based authentication and key exchange protocols into two categories as follows:

- *The PUF-based authentication and key exchange protocols using public key algorithms.* The public key algorithms have high key management flexibility. This is because the communicating parties have two different keys, one of which is kept as a secret and the other is made public. There is no need to share the secret key as in symmetric key algorithms. The PUF-based protocols using public key algorithms inherit this feature. Yilmaz et al. [5] applied the RSA algorithm in the lightweight PUF-based authentication protocol. Chatterjee et al. [6] developed an authentication and key exchange protocol combining PUFs, identity-based encryption (IBE), and the keyed hash function. Chuang et al. [7] designed a PUF-based authenticated key exchange protocol for IoT devices without verifiers and an explicit challenge–response pair (CRP). Using certificateless public key cryptography (CL-PKC), Li et al. [8] proposed a PUF-based end-to-end mutual authentication and key exchange protocol for IoT devices. Chaterjee et al. [9] proposed a private PUF-based anonymous authentication protocol. Siddiqui et al. [10] proposed a PUF-based authentication protocol, which is dependent on two certificate authorities of the cloud IoT system. Harishma et al. [11] proposed a PUF-based operationally asymmetric mutual authentication and key exchange protocol for secure communication. Qureshi and Munir [12] introduced a lightweight CRP obfuscation mechanism using XOR and shuffle operations and further used it to design the PUF-based key exchange protocol.
- *The PUF-based authentication and key exchange protocols using symmetric key algorithms.* The advantage of symmetric key algorithms is low computational cost. These algorithms are therefore well suited for authentication and key exchange protocols for resource-constrained IoT devices and help to reduce the computational burden of these IoT devices. In the PUF-based protocols using symmetric key algorithms, shared keys are generated by PUFs and hash functions are commonly used to authenticate messages. The researchers [13,14] proposed PUF-based mutual authentication protocols for IoT systems. Their protocols are presented for two scenarios, that is, device-to-device communication and device-to-server communication. Qureshi and Munir [15] presented a PUF-based identity-preserving protocol. During the authentication run of their protocol, the server does not store, generate, transmit, or receive complete devices’ CRPs. Lounis and Zulkernine [16] proposed PUF-based Thing-to-Thing (T2T) architectures, where devices autonomously authenticate each other without any human intervention involved. Nimmy et al. [17] proposed a protocol that avoids explicit storage of CRPs for verification by using geometric threshold secret sharing. Zheng and Chang [18] proposed a new lightweight PUF-based mutual authentication and key exchange protocol for two PUF-embedded IoT devices. Wang et al. [19] leveraged PUFs to generate symmetric shared keys in the lightweight protocol. Ebrahimabadi et al. [20] designed an authentication protocol to thwart modeling attacks for PUF-based IoT devices. Zerrouki et al. [21] proposed a mutual authentication and session key establishment protocol for IoT devices based on PUFs. Sun and Tian [22] further gave security analysis and improvements to this protocol and suggested the idea of a key compromise to evaluate other novel PUF protocol designs. Wang et al. [23] introduced a supplementary sub-protocol to the PUF-based authentication protocol for the purpose of enhancing resistance to desynchronization attacks. Park and Park [24] employed PUFs to realize an improved authentication and key agreement protocol for cloud-enabled IoT devices. To achieve decentralization and security, Aseeri et al. [25] introduced a secure, lightweight, cost-efficient reinforcement machine learning framework (SLCR-MLF) with PUFs. In addition, some researchers [26,27,28] proposed PUF-based two-factor authentication mechanisms.

### 1.2. Our Motivations and Contributions

In *IEEE Internet Things J.* 2023, 10, 8547–8559, Roy et al. [29] proposed a PUF-based authentication and key exchange protocol. Roy et al.’s protocol is interesting because it tries to leverage cryptographic XOR operation and hash function for traditional secure communication and PUF for preventing physical attacks. Moreover, this standalone protocol can perform device-to-device and device-to-server authentication, eradicating the need for disparate protocols. In addition, using the Scyther verifier tool, the security of Roy et al.’s protocol is evaluated by the formal method.

Due to their inherent vulnerability, IoT devices (nodes) are easily compromised by insider attackers. Therefore, in this paper, we carefully study the security of Roy et al.’s protocol under the assumption of an inside attack. Our results are summarized as follows:

(1) A legitimate but malicious IoT node can monitor the secure communication among the server and any other IoT nodes, because the malicious IoT node can reveal their secret session key. The only requirement of the malicious IoT node is to observe the transmitted messages over public channel during the authentication and key exchange phase of Roy et al.’s protocol.
(2) A legitimate but malicious IoT node is able to impersonate a target IoT node to cheat the server and any other IoT nodes during the authentication and key exchange phase of Roy et al.’s protocol. Finally, the malicious IoT node can establish a secret session key for the subsequent secure communication.
(3) A legitimate but malicious IoT node can masquerade as the server to cheat any other target IoT nodes during the authentication and key exchange phase of Roy et al.’s protocol. Here, the malicious IoT node is able to generate the valid session key for the target IoT nodes.

## 2. Basic Knowledge of PUF

PUFs make use of the intrinsic random variability in the physical microstructure of integrated circuits (ICs) to produce a unique *n*-bit response *R_i_* to an *m*-bit challenge *C_i_* for any *i*∈N. We can write a PUF’s instance as follows.

*f_PUF_*: *C_i_*→*R_i_*, (1)

where *C_i_*∈{0,1}*^m^* and *R_i_*∈{0,1}*^n^*.

PUF has the following safety features:

- Uniqueness: PUFs cannot output the same response for different input challenges, while different PUFs output different responses for the same input challenge. For two different *C_i_* and *C_j_*, with *i*, *j*∈N, and any two different PUF instances *f^A^_PUF_* and *f^B^_PUF_*, the uniqueness expression is as follows.
  *f^A^_PUF_*(*C_i_*) ≠ *f^A^_PUF_*(*C_j_*). (2)
  *f^A^_PUF_*(*C_i_*) ≠ *f^B^_PUF_* (*C_i_*). (3)
- Reliability: This feature measures the reproducibility of a PUF response under different operating conditions for a given challenge. The reliability expression of the PUF instance for different time periods *t*_1_ and *t*_2_ is:
  *f_PUF_*(*C_i_*)|*_t_*_= *t*_1__ = *f_PUF_*(*C_i_*)|*_t_*_= *t*_2__. (4)
  In fact, the PUF response is sensitive to various noise elements, such as temperature, voltage, and other environmental changes. Under the same challenge, the noise can cause the PUF to give an erroneous response, which is different from the original one. To eliminate the effects of such noise, Roy et al. [29] considered the use of lightweight error-correcting algorithms (ECAs) to stabilize the noisy PUF response and thus improved its reliability.

## 3. Review of Roy et al.’s Protocol

The one-time enrollment phase is responsible for enrolling the new IoT nodes in the server. The authentication and key exchange phase realizes mutual authentication between the server and the node(s). To maintain consistency, we employ the same notions of [29] and write them in Table 1.

### 3.1. One-Time Enrollment Phase

This phase is run in a secure environment without the attacker. After completing this phase, the IoT nodes can be deployed in the IoT network. When any *A* wants to enroll in the server, the server randomly generates an input *C_Ai_* (i.e., challenge) for *A*’s PUF and collects its output *R_Ai_* (i.e., response), where *R_Ai_* = *f^A^_PUF_*(*C_Ai_*). Then, the server stores *A*’s ID-CRP {*id_A_*, *C_Ai_*, *R_Ai_*} in its secure database. The server only maintains one ID-CRP record for each IoT device.

### 3.2. Authentication and Key Exchange Phase

This phase has two communication models, i.e., Node-to-Node (N2N) communication and Node-to-Server (N2S) communication. N2N communication aims to provide the mutual authentication and key establishment of any two IoT nodes. Meanwhile, N2S communication realizes the mutual authentication and key establishment between any one IoT node and the server.

#### 3.2.1. N2N Communication

**Figure 2:**
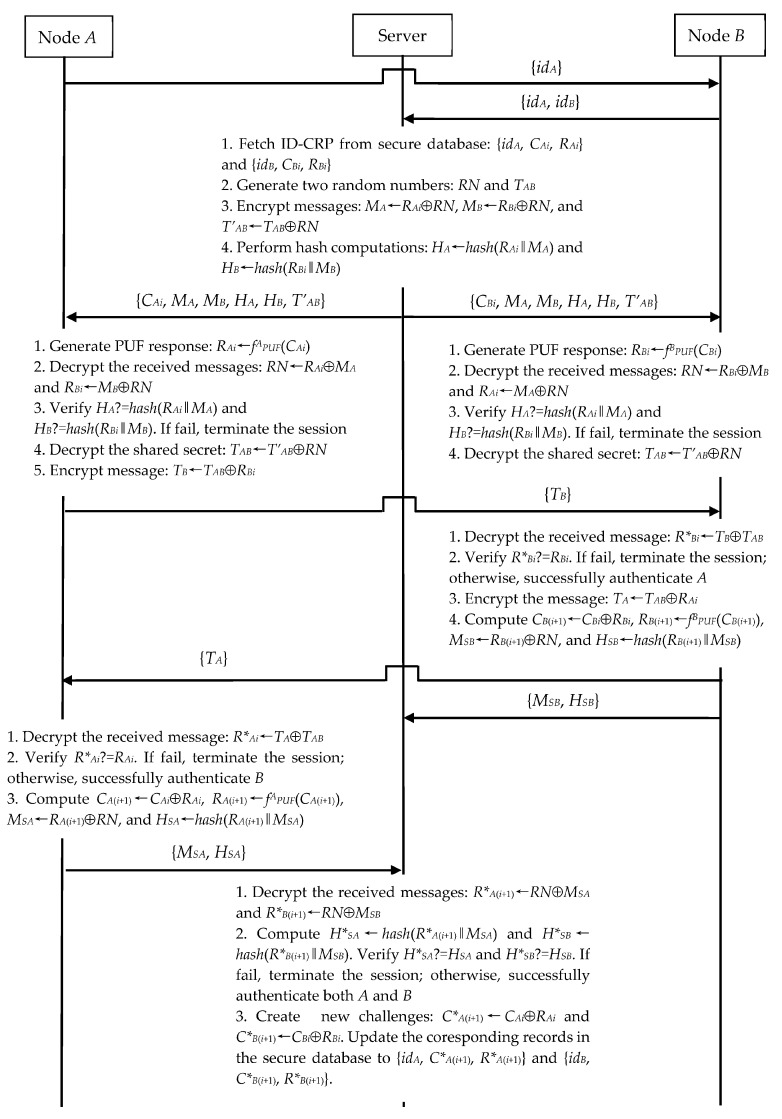
N2N’s authentication and key exchange phase.

Assume that two nearby *A* and *B* nodes want to authenticate each other and establish their session key. As shown in Figure 2, *A*, *B*, and the server perform the following steps.

Step 1. *A* sends a connection request {*id_A_*} to *B*. Upon receiving this request, *B* sends the N2N connection establishment request {*id_A_*, *id_B_*} to the server.

Step 2. The server fetches corresponding ID-CRPs {*id_A_*, *C_Ai_*, *R_Ai_*} and {*id_B_*, *C_Bi_*, *R_Bi_*} from its secure database and generates two random numbers *RN* and *T_AB_*. The server computes:

*M_A_*←*R_Ai_*⊕*RN* (5)

*M_B_*←*R_Bi_*⊕*RN* (6)

*T’_AB_*←*T_AB_*⊕*RN* (7)

*H_A_*←*hash*(*R_Ai_*‖*M_A_*) (8)

*H_B_*←*hash*(*R_Bi_*‖*M_B_*). (9)

Then, the server sends the message {*C_Ai_*, *M_A_*, *M_B_*, *H_A_*, *H_B_*, *T’_AB_*} to *A* and the message {*C_Bi_*, *M_A_*, *M_B_*, *H_A_*, *H_B_*, *T’_AB_*} to *B*.

Step 3. Upon receiving the message {*C_Ai_*, *M_A_*, *M_B_*, *H_A_*, *H_B_*, *T’_AB_*}, *A* first computes:

*R_Ai_*←*f^A^_PUF_*(*C_Ai_*) (10)

*RN*←*R_Ai_*⊕*M_A_* (11)

*R_Bi_*←*M_B_*⊕*RN* (12)

H*_A_←*hash*(*R_Ai_*‖*M_A_*) (13)

H*_B_←*hash*(*R_Bi_*‖*M_B_*). (14)

To authenticate the server, *A* then checks whether *H_A_* is equal to H*_A_ and *H_B_* is equal to H*_B_. If any verification fails, *A* terminates the session. Otherwise, *A* further calculates:

*T_AB_*←*T’_AB_*⊕*RN* (15)

*T_B_*←*T_AB_*⊕*R_Bi_*. (16)

Finally, *A* sends {*T_B_*} to *B*.

Step 4. Upon receiving the messages {*C_Bi_*, *M_A_*, *M_B_*, *H_A_*, *H_B_*, *T’_AB_*} and {*T_B_*}, *B* computes:

*R_Bi_*←*f^B^_PUF_*(*C_Bi_*) (17)

*RN*←*R_Bi_*⊕*M_B_* (18)

*R_Ai_*←*M_A_*⊕*RN* (19)

H*_A_←*hash*(*R_Ai_*‖*M_A_*) (20)

H*_B_←*hash*(*R_Bi_*‖*M_B_*). (21)

To authenticate the server, *B* checks *H_A_* and *H_B_* using H*_A_ and H*_B_ just like *A*. If any verification fails, *B* also terminates the session. Moreover, *B* evaluates:

*T_AB_*←*T’_AB_*⊕*RN* (22)

R*_Bi_←*T_B_*⊕*T_AB_*. (23)

Now, *B* checks whether *R_Bi_* is equal to R*_Bi_. If the verification is incorrect, *B* terminates the session; otherwise, *B* computes:

*T_A_*←*T_AB_*⊕*R_Ai_* (24)

*C_B_*_(*i*+1)_←*C_Bi_*⊕*R_B__i_* (25)

*R_B_*_(*i*+1)_ ←*f^B^_PUF_*(*C_B_*_(*i*+1)_) (26)

*M_SB_*←*R_B_*_(*i*+1)_⊕*RN* (27)

*H_SB_*←*hash*(*R_B_*_(*i*+1)_‖*M_SB_*). (28)

Finally, *B* sends {*T_A_*} to *A* and {*M_SB_*, *H_SB_*} to the server.

Step 5. Upon receiving the message {*T_A_*}, *A* computes:

R*_Ai_←*T_A_*⊕*T_AB_*. (29)

*A* checks whether *R_Ai_* is equal to R*_Ai_. If the verification is incorrect, *A* terminates the session; otherwise, *A* computes:

*C_A_*_(*i*+1)_←*C_Ai_*⊕*R_Ai_* (30)

*R_A_*_(*i*+1)_←*f^A^_PUF_*(*C_A_*_(*i*+1)_) (31)

*M_SA_*←*R_A_*_(*i*+1)_⊕*RN* (32)

*H_SA_*←*hash*(*R_A_*_(*i*+1)_‖*M_SA_*). (33)

*A* then sends {*M_SA_*, *H_SA_*} to the server.

Step 6. Upon receiving the messages {*M_SA_*, *H_SA_*} and {*M_SB_*, *H_SB_*}, the server computes:

R*_A__(*i*+1)_←*RN*⊕*M_SA_* (34)

R*_B__(*i*+1)_←*RN*⊕*M_SB_* (35)

H*_SA_←*hash*(R*_A__(*i*+1)_‖*M_SA_*) (36)

H*_SB_←*hash*(R*_B__(i+1)_‖*M_SB_*). (37)

The server then checks whether *H_SA_* is equal to H*_SA_ and *H_SB_* is equal to H*_SB_. If any verification is incorrect, the server terminates the session; otherwise, the server computes:

C*_A__(*i*+1)_←*C_Ai_*⊕*R_Ai_* (38)

C*_B__(*i*+1)_←*C_Bi_*⊕*R_Bi_*. (39)

Now, the server updates {*id_A_*, *C_Ai_*, *R_Ai_*} to {*id_A_*, C*_A__(*i*+1)_, R*_A__(*i*+1)_} and {*id_B_*, *C_Bi_*, *R_Bi_*} to {*id_B_*, C*_B__(*i*+1)_, R*_B__(*i*+1)_} in its secure database.

After the successful completion of above steps, *T_AB_* is used as the secret session key for subsequent secure communication between *A* and *B*. Clearly, *T_AB_* gets updated in every new session.

#### 3.2.2. N2S Communication

**Figure 3:**
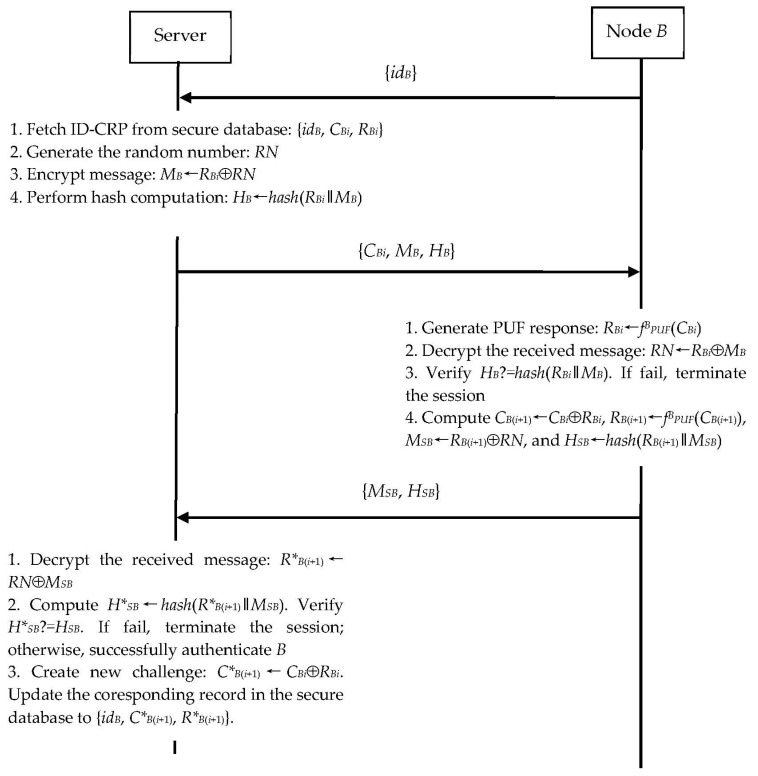
N2S’s authentication and key exchange phase.

Assume that *B* wants to authenticate the proximity server and establish a session key with the server. As shown in Figure 3, *B* and the server perform the following steps.

Step 1. *B* sends the N2S connection establishment request {*id_B_*} to the server.

Step 2. The server fetches the corresponding ID-CRP {*id_B_*, *C_Bi_*, *R_Bi_*} from its secure database and generates a random number *RN*. The server computes:

*M_B_*←*R_Bi_*⊕*RN* (40)

*H_B_*←*hash*(*R_Bi_*‖*M_B_*). (41)

The server then sends the message {*C_Bi_*, *M_B_*, *H_B_*} to *B*.

Step 3. Upon receiving the message {*C_Bi_*, *M_B_*, *H_B_*}, *B* computes:

*R_Bi_*←*f^B^_PUF_*(*C_Bi_*) (42)

*RN*←*R_Bi_*⊕*M_B_* (43)

H*_B_←*hash*(*R_Bi_*‖*M_B_*). (44)

To authenticate the server, *B* then checks whether *H_B_* is equal to H*_B_. If any verification fails, *B* terminates the session. Otherwise, *B* further computes:

*C_B_*_(*i*+1)_←*C_Bi_*⊕*R_B__i_* (45)

*R_B_*_(*i*+1)_←*f^B^_PUF_*(*C_B_*_(*i*+1)_) (46)

*M_SB_*←*R_B_*_(*i*+1)_⊕*RN* (47)

*H_SB_*←*hash*(*R_B_*_(*i*+1)_‖*M_SB_*). (48)

Finally, *B* sends {*M_SB_*, *H_SB_*} to the server.

Step 4. Upon receiving the message {*M_SB_*, *H_SB_*}, the server computes:

R*_B__(*i*+1)_ ←*RN*⊕*M_SB_* (49)

H*_SB_←*hash*(R*_B__(*i*+1)_‖*M_SB_*). (50)

The server then checks whether *H_SB_* is equal to H*_SB_. If the verification is incorrect, the server terminates the session; otherwise, the server computes:

C*_B__(*i*+1)_←*C_Bi_*⊕*R_Bi_*. (51)

Now, the server updates {*id_B_*, *C_Bi_*, *R_Bi_*} to {*id_B_*, C*_B__(*i*+1)_, R*_B__(*i*+1)_} in its secure database.

Once the above authentication procedure is completed, *B* and the server share *R_B_*_(*i*+1)_ as their secret session key. This key is updated in every new session establishment.

## 4. Insider Attack on Roy et al.’s Protocol

Let *A* be an insider attacker. It means that *A* has a legitimate ID-CRP record {*id_A_*, *C_Ai_*, *R_Ai_*} in the server’s secure database but attempts to sabotage the server and other IoT nodes. When both *A* and *B* run the N2N communication model with the server, *A* executes the steps as described in Figure 2. At the same time, *A* as an insider further performs the following operations.

(1) *A* eavesdrops on *B*’s {*id_A_*, *id_B_*} during Step 1.
(2) *A* eavesdrops on the server’s {*C_Bi_*, *M_A_*, *M_B_*, *H_A_*, *H_B_*, *T’_AB_*} during Step 2.
(3) In Step 4, *A* eavesdrops on *B*’s {*M_SB_*, *H_SB_*}.

Based on above, *A* can compute *B*’s *C_B_*_(*i*+1)_ by evaluating *C_Bi_*⊕*R_Bi_*, because it knows *R_Bi_* (see Equation (12)) during Step 3. And *A* can correctly recover *B*’s *R_B_*_(*i*+1)_ by calculating *M_SB_*⊕*RN*. Here, we know that *A* also computes the correct *RN* (see Equation (11)) during Step 3. Finally, we conclude that *A* obtains *B*’s {*id_B_*, *C_Bi_*, *R_Bi_*} used in the next session, i.e., {*id_B_*, *C_B_*_(*i*+1)_, *R_B_*_(*i*+1)_}.

### 4.1. Surveillance on IoT Nodes and Server

#### 4.1.1. Surveillance Exploiting N2N Communication

**Figure 4:**
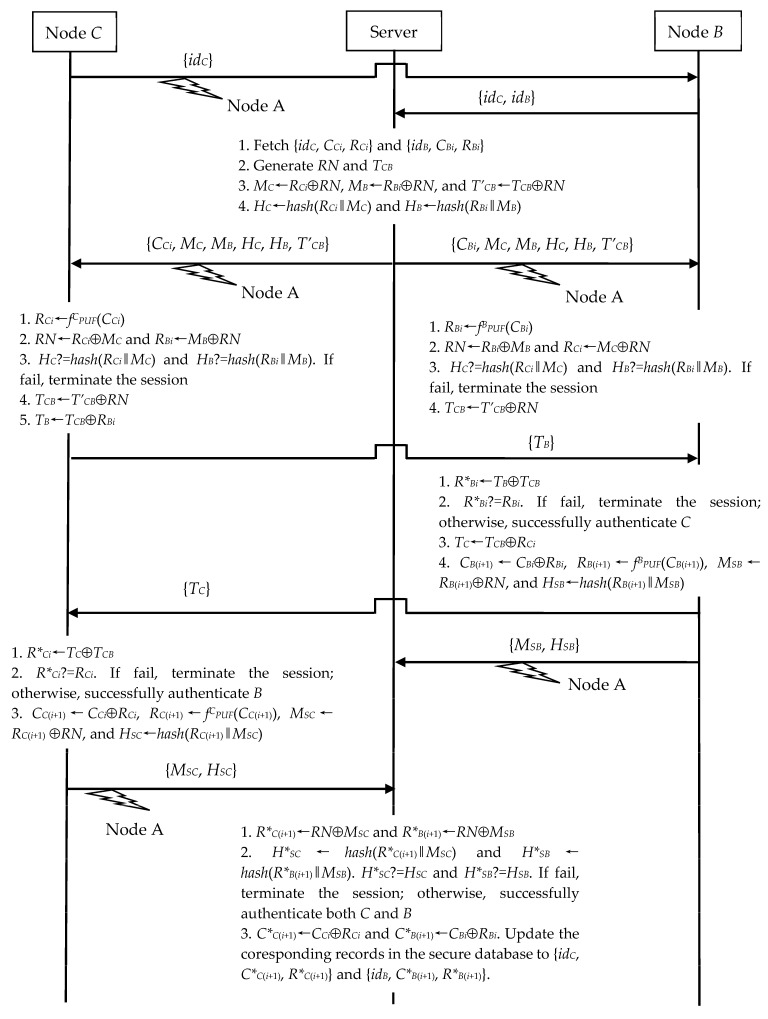
Eavesdropping on N2N’s authentication and key exchange phase.

To achieve authentication and establish the session key, *B* runs the N2N communication model with any *C*. As shown in Figure 4, *A* can eavesdrop on their session messages. And then, *A* further discloses the secret session key *T_CB_* between *C* and *B*. Hence, *A* can monitor the subsequent secret channel using *T_CB_*. For more detail, we describe *A*’s behaviors on the session run of the N2N communication model between *C* and *B*.

(1) In Step 1, *A* eavesdrops on *C*’s {*id_c_*}.
(2) In Step 2, *A* eavesdrops on the server’s {*C_Bi_*, *M_C_*, *M_B_*, *H_C_*, *H_B_*, *T’_CB_*}.

Now, *A* can obtain the *RN* by computing *R_Bi_*⊕*M_B_*, where *R_Bi_* is collected in *A*’s previous session with *B*. *A* then recovers *T_CB_* by computing *T’_CB_*⊕*RN*. Moreover, if *A* wants to monitor *B*’s next session, it can further perform as follows.

In Step 4, *A* eavesdrops on *B*’s {*M_SB_*, *H_SB_*}.

*A* can compute *B*’s *C_B_*_(*i*+1)_ by evaluating *C_Bi_*⊕*R_Bi_* and then recover *B*’s *R_B_*_(*i*+1)_ by calculating *M_SB_*⊕*RN*. In the end, *A* updates its {*id_B_*, *C_Bi_*, *R_Bi_*} to {*id_B_*, *C_B_*_(*i*+1)_, *R_B_*_(*i*+1)_} for *B*’s next session. In addition, if *A* wants to monitor *C*’s next session, it further performs in the following.

(1) In Step 2, *A* eavesdrops on the server’s {*C_Ci_*, *M_C_*, *M_B_*, *H_C_*, *H_B_*, *T’_CB_*}.
(2) In Step 5, *A* eavesdrops on *C*’s {*M_SC_*, *H_SC_*}.

In this situation, *A* computes *R_Ci_* by using *M_C_*⊕*RN* and *C_C_*_(*i*+1)_ by using *C_Ci_*⊕*R_Ci_*. *A* also recovers *C*’s *R_B_*_(*i*+1)_ by calculating *M_SC_*⊕*RN*. Finally, *A* can record {*id_C_*, *C_C_*_(*i*+1)_, *R_C_*_(*i*+1)_} for monitoring *C*’s next session.

*Comments*. According to above surveillance, in the initial stage, the insider attacker needs to collect information about the target IoT node (i.e., *B*) by running a session with the target IoT node. Afterwards, the insider attacker can expand their surveillance to other IoT nodes (e.g., *C*), which run the sessions with the target IoT node. Meanwhile, the insider attacker no longer needs to participate in those session runs and still obtains those secrets. This effectively hides the insider attacker and reduces the probability of the insider attacker being identified. Moreover, the insider attacker can establish a surveillance network of the selected IoT nodes based on our proposed attack.

#### 4.1.2. Surveillance Exploiting N2S Communication

**Figure 5:**
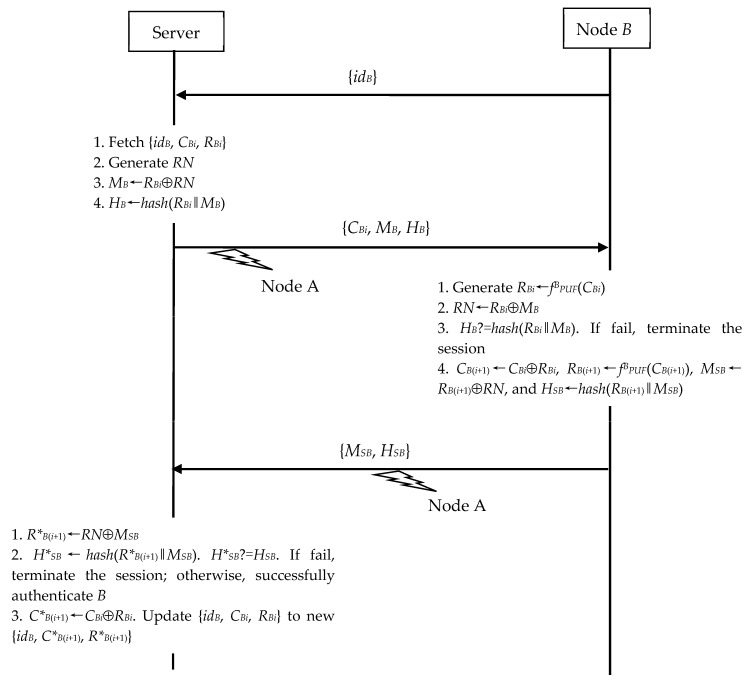
Eavesdropping on N2S’s authentication and key exchange phase.

*A* is able to monitor the session run of the N2S communication model between *B* and the server. As shown in Figure 5, *A* can perform the following operations to reveal the secret session key *R_B_*_(*i*+1)_ shared by *B* and the server.

(1) In Step 2, *A* eavesdrops on the server’s {*C_Bi_*, *M_B_*, *H_B_*}.
(2) In Step 3, *A* eavesdrops on *B*’s {*M_SB_*, *H_SB_*}.

Because *A* knows *B*’s *R_Bi_*, *A* can obtain the *RN* by computing *R_Bi_*⊕*M_B_*. Now, *A* further recovers the secret session key *R_B_*_(*i*+1)_ by computing *M_SB_*⊕*RN*. *A* can therefore observe the following secure communication using *R_B_*_(*i*+1)_. Moreover, if *A* wants to continuously monitor the session run of the N2S communication model between *B* and the server, *A* merely computes *C_B_*_(*i*+1)_ = *C_Bi_*⊕*R_Bi_* and replaces the old {*id_B_*, *C_Bi_*, *R_Bi_*} with the new {*id_B_*, *C_B_*_(*i*+1)_, *R_B_*_(*i*+1)_}.

### 4.2. Impersonating IoT Node

#### 4.2.1. IoT Node Impersonation Exploiting N2N Communication

**Figure 6:**
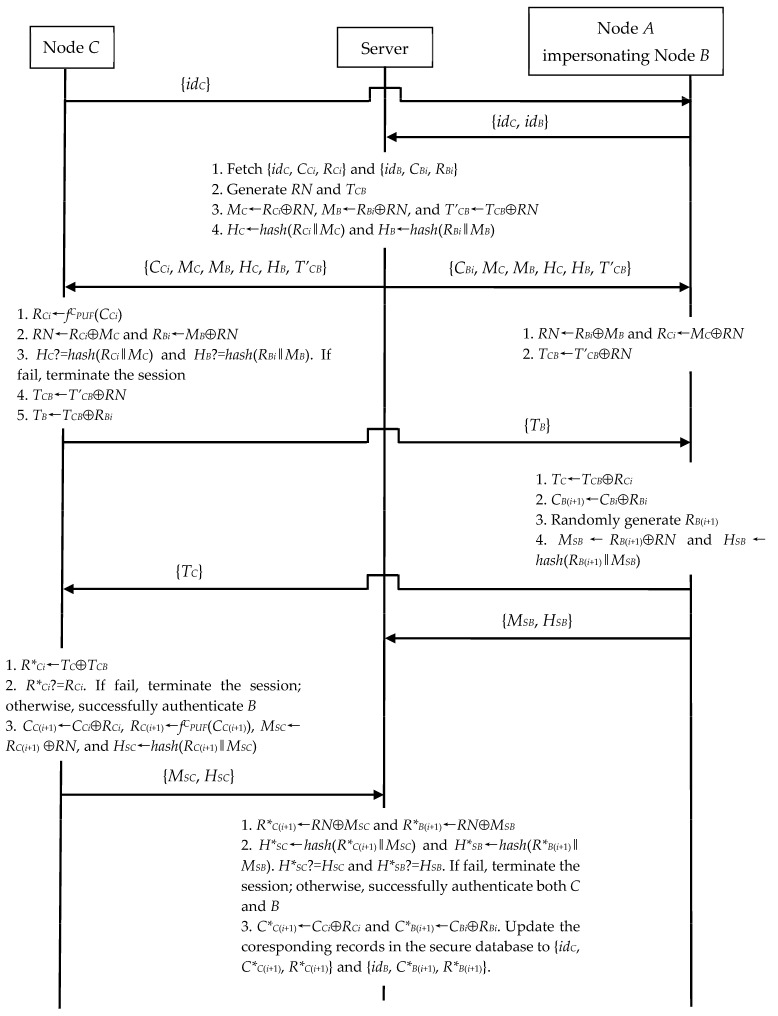
Node impersonation during N2N’s authentication and key exchange phase.

In N2N communication, *A* can imitate *B* run with any *C*, when *A* obtains a legal {*id_B_*, *C_Bi_*, *R_Bi_*}. As shown in Figure 6, *A* replaces *B* to perform *B*’s following steps during the run of N2N communication.

(1) Upon receiving *C*’s request {*id_C_*} in Step 1, *A* sends the N2N connection establishment request {*id_C_*, *id_B_*} to the server.
(2) Upon receiving the messages {*C_Bi_*, *M_C_*, *M_B_*, *H_C_*, *H_B_*, *T’_CB_*} and {*T_B_*} in Step 4, *A* computes *RN*←*R_Bi_*⊕*M_B_*, *R_Ci_*←*M_C_*⊕*RN*, *T_CB_*←*T’_CB_*⊕*RN*, *T_C_*←*T_CB_*⊕*R_Ci_*, and *C_B_*_(*i*+1)_←*C_Bi_*⊕*R_Bi_*. Then, *A* generates *R_B_*_(*i*+1)_ at random. Next, *A* evaluates *M_SB_*←*R_B_*_(*i*+1)_⊕*RN* and *H_SB_*←*hash*(*R_B_*_(*i*+1)_‖*M_SB_*). Finally, *A* sends {*T_C_*} to *C* and {*M_SB_*, *H_SB_*} to the server.

Herein, *A* randomly generates *R_B_*_(*i*+1)_ instead of *B*’s *R_B_*_(*i*+1)_ outputted by the valid PUF to evaluate *M_SB_* and *H_SB_* in Step 4. *A* can still pass the server’s authentication, because the server never checks the PUF validness of its receiving *R_B_*_(*i*+1)_ in Step 6. If *A* wants to continuously impersonate *B*, *A* further uses the new {*id_B_*, *C_B_*_(*i*+1)_, *R_B_*_(*i*+1)_} to replace the old {*id_B_*, *C_Bi_*, *R_Bi_*} for *B*’s next session. In addition, *A* can also employ a similar attack to impersonate initiator *C*, if *A* obtains *C*’s {*id_C_*, *C_Ci_*, *R_Ci_*}.

*Comments.* After *A* completes above impersonation, *B* will no longer be able to successfully run the authentication and key exchange phase with the server and other IoT nodes. In this situation, we know that the server updates its {*id_B_*, *C_Bi_*, *R_Bi_*} to {*id_B_*, *C_B_*_(*i*+1)_, *R_B_*_(*i*+1)_}, where *R_B_*_(*i*+1)_ is randomly generated by *A*. When *B* runs the authentication and key exchange phase, the server should use the random *R_Bi_* to generate *M_B_* (see Equation (6)) and send it to *B* in Step 2. However, in Step 4, *B* retrieves its local *R_Bi_* by invoking *f^B^_PUF_*(*C_Bi_*) (see Equation (17)). Clearly, the server’s *R_Bi_* and *B*’s *R_Bi_* are always not equal because of the PUF’s safety feature. Hence, *B* should recover an incorrect *RN* using Equation (18) and fail the subsequent authentication and key exchange procedure with the server and the counterpart IoT node. Therefore, *B* suffers from a denial of service attack due to *A*’s impersonation.

#### 4.2.2. IoT Node Impersonation Exploiting N2S Communication

**Figure 7:**
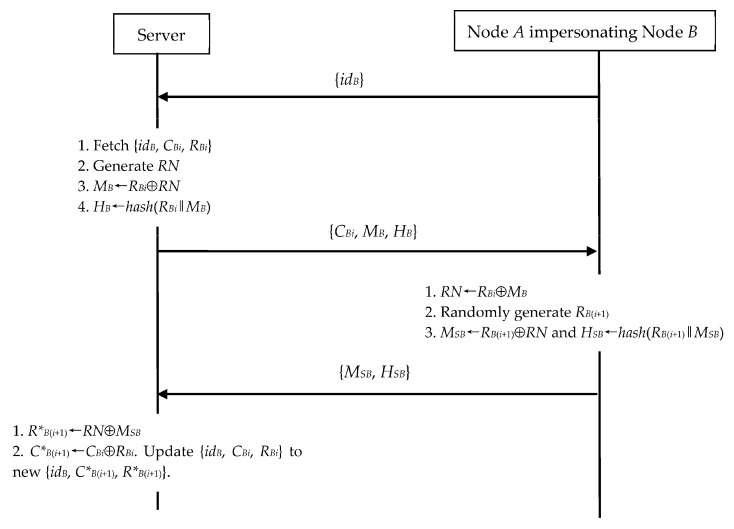
Node impersonation during N2S’s authentication and key exchange phase.

In N2S communication, *A* can impersonate *B* to cheat the server, when *A* obtains *B*’s {*id_B_*, *C_Bi_*, *R_Bi_*}. As shown in Figure 7, we demonstrate *A*’s operations as follows.

(1) *A* sends the request {*id_B_*} to the server in Step 1.
(2) Upon receiving the message {*C_Bi_*, *M_B_*, *H_B_*} in Step 3, *A* computes *RN*←*R_Bi_*⊕*M_B_*. Then, *A* generates *R_B_*_(*i*+1)_ at random. Next, *A* evaluates *M_SB_*←*R_B_*_(*i*+1)_⊕*RN* and *H_SB_*←*hash*(*R_B_*_(*i*+1)_‖*M_SB_*). Finally, *A* sends {*M_SB_*, *H_SB_*} to the server.

This node impersonation is similar to that of the impersonation discussed in Section 4.2.1. Clearly, the server should confirm the validity of *A*’s *H_SB_* in Step 4, and therefore we omit this operation in Figure 7 for simplicity. The slight difference is that, after session run, *A* applies *R_B_*_(*i*+1)_ as the secret session key instead of *T_CB_* as in Section 4.2.1.

### 4.3. Impersonating Server

#### 4.3.1. Server Impersonation Exploiting N2N Communication

**Figure 8:**
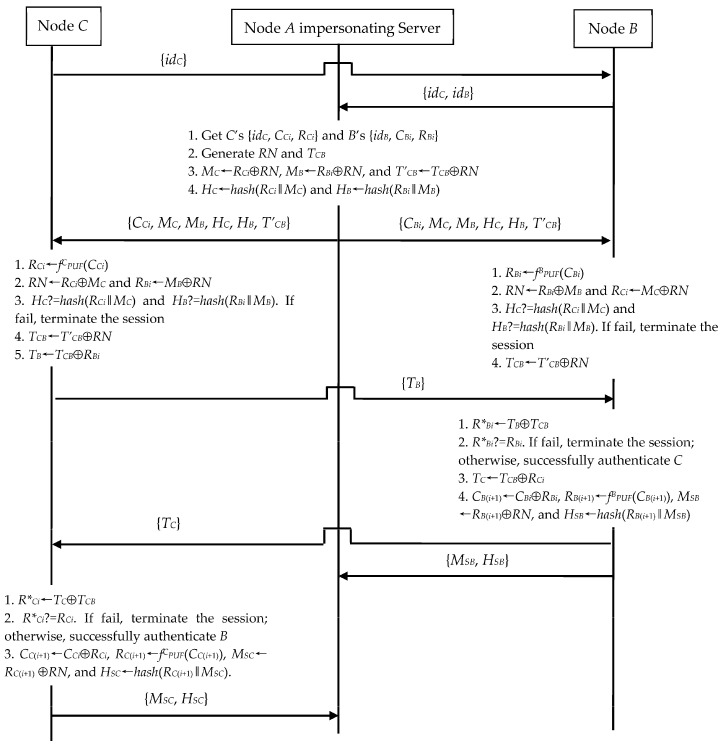
Server impersonation during N2N’s authentication and key exchange phase.

*A* can impersonate the server to cheat both *C* and *B* in N2N communication, if *A* obtains *C*’s {*id_C_*, *C_Ci_*, *R_Ci_*} and *B*’s {*id_B_*, *C_Bi_*, *R_Bi_*}. Figure 8 shows the process of *A*’s server impersonation. *A* performs the following operations.

(1) In Step 2, *A* randomly generates *RN* and *T_CB_*. *A* further computes *M_C_*←*R_Ci_*⊕*RN*, *M_B_*←*R_Bi_*⊕*RN*, *T’_CB_*←*T_CB_*⊕*RN*, *H_C_*←*hash*(*R_Ci_*‖*M_C_*), and *H_B_*←*hash*(*R_Bi_*‖*M_B_*). Then, *A* sends the message {*C_Ci_*, *M_C_*, *M_B_*, *H_C_ H_B_*, *T’_CB_*} to *C* and the message {*C_Bi_*, *M_C_*, *M_B_*, *H_C_*, *H_B_*, *T’_CB_*} to *B*.
(2) Upon receiving the messages {*M_SC_*, *H_SC_*} and {*M_SB_*, *H_SB_*} in Step 6, *A* omits them.

*A* finally shares the session key *T_CB_* with both *C* and *B*. Moreover, *A* still can reuse *C*’s {*id_C_*, *C_Ci_*, *R_Ci_*} and *B*’s {*id_B_*, *C_Bi_*, *R_Bi_*} to impersonate the server in the subsequent session, because *C* and *B* do not verify the freshness of *C_Ci_* and *C_Bi_* in each session run. Certainly, in Step 6, *A* can also compute R*_C__(*i*+1)_←*RN*⊕*M_SC_*, R*_B__(*i*+1)_←*RN*⊕*M_SB_*, C*_C__(*i*+1)_←*C_Ci_*⊕*R_Ci_*, and C*_B__(*i*+1)_←*C_Bi_*⊕*R_Bi_*. This means that *A* obtains new ID-CRPs for future attacks.

#### 4.3.2. Server Impersonation Exploiting N2S Communication

**Figure 9:**
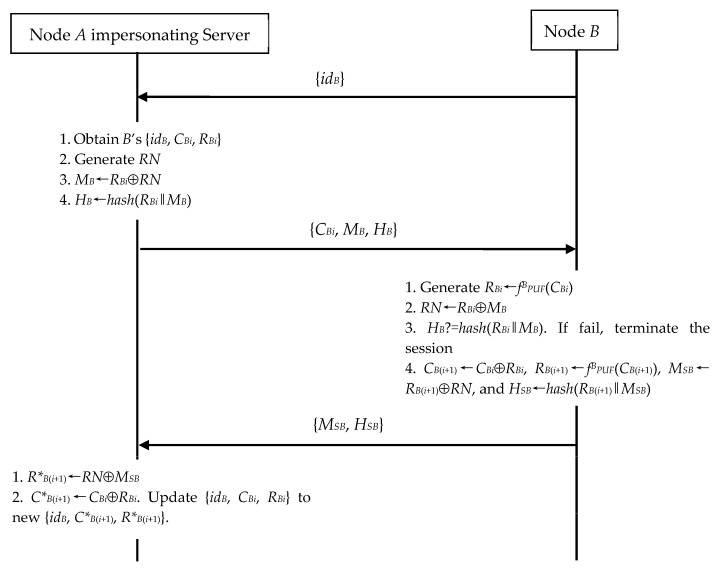
Server impersonation during N2S’s authentication and key exchange phase.

When *B* wants to run N2S communication, *A* can make use of *B*’s {*id_B_*, *C_Bi_*, *R_Bi_*} to impersonate the server. As shown in Figure 9, *A* executes the following operations to achieve it.

(1) In Step 2, *A* generates the random number *RN*. *A* computes *M_B_*←*R_Bi_*⊕*RN* and *H_B_*←*hash*(*R_Bi_*‖*M_B_*). Then, *A* sends the message {*C_Bi_*, *M_B_*, *H_B_*} to *B*.
(2) Upon receiving the message {*M_SB_*, *H_SB_*} in Step 4, *A* computes R*_B__(*i*+1)_←*RN*⊕*M_SB_* and C*_B__(*i*+1)_←*C_Bi_*⊕*R_Bi_* and further replaces its {*id_B_*, *C_Bi_*, *R_Bi_*} with {*id_B_*, C*_B__(*i*+1)_, R*_B__(*i*+1)_}.

As a result, *A* and *B* establish the secret session key R*_B__(*i*+1)_. *A* can omit to replace its {*id_B_*, *C_Bi_*, *R_Bi_*} with {*id_B_*, C*_B__(*i*+1)_, R*_B__(*i*+1)_} and still use {*id_B_*, *C_Bi_*, *R_Bi_*} for the subsequent impersonation. The reason is the same as in our discussion in Section 4.3.1.

### 4.4. Discussion of Insider Attacker

We discuss how an IoT node practically becomes a malicious IoT node, i.e., an insider attacker. In general, the IoT system contains plenty of IoT nodes. And some of them are possibly manufactured and provided by third parties. From a third-party perspective, these IoT nodes may not only want to work properly in the IoT system, but also engage in malicious behaviors such as surveillance and impersonation. Clearly, in this situation, the malicious IoT nodes can enter the IoT system during the one-time enrollment phase of Roy et al.’s protocol.

After the deployment of the IoT nodes, physical attacks are very common for the IoT system. They typically require physical proximity to the IoT system and can involve actions that limit the efficacy of the IoT nodes. In order to turn benign IoT nodes into malicious IoT nodes, the physical attack can further inject malicious codes into benign IoT nodes. These malicious codes include the logic that requires the IoT nodes to execute insider attacks in some cases. In addition, software attacks can be exploited to compromise the IoT nodes, that is, the attackers use the malware, such as viruses, worms, and Trojans, to manipulate the IoT nodes.

It is possible that malicious IoT nodes have limited resources and functionalities. This means that the malicious IoT nodes cannot handle and store the derived session keys for next session and for another IoT node, cannot intercept the transmitted messages over the public channel, cannot impersonate the server, and so on. To complete our insider attacks, the attacker can build a powerful auxiliary node to support malicious IoT nodes. Each malicious IoT node *A* merely outputs its {*id_A_*, *C_Ai_*, *R_Ai_*} to the auxiliary node. The auxiliary node can replace the malicious IoT node *A* to implement the insider attacks.

## 5. Experimental Verification of Proposed Insider Attacks

Scyther is a formal analysis tool for automatic verification of security protocols based on the security protocol description language (SPDL). Scyther can analyze protocols that contain multiple subjects, infinite session interaction, and the use of random numbers. We therefore use Scyther to confirm our insider attacks on Roy et al.’s protocol. We implement experimental verification on the 64-bit Windows 10 operating system, Graphviz v2.50, and Python v2.7 using the Compile-0.9.2 version of the Scyther tool.

When using the Scyther tool to verify Roy et al.’s protocol, we write secret PUF responses, XOR-encrypted messages, etc., into the SPDL script to model the protocol. The details of the Scyther tool parameter settings are shown in Table 2. Here, the attacker can obtain the secret PUF responses and furthermore derive the session key and random numbers. This is consistent with the capabilities of the malicious IoT nodes assumed in Section 4.

**Figure 10:**
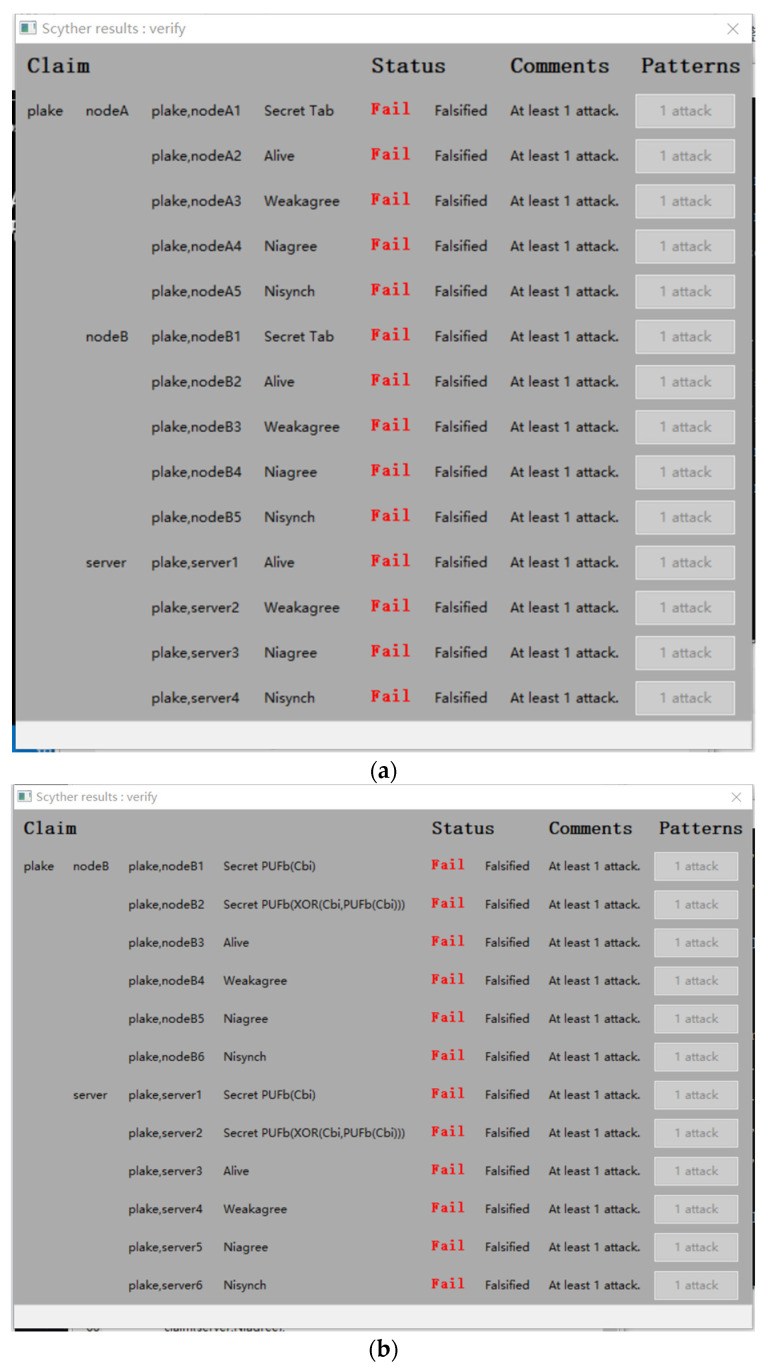
Scyther outputs for Roy et al.’s protocol. (**a**) N2N communication; (**b**) N2S communication.

For Roy et al.’s protocol, Figure 10 shows Scyther’s verification results for N2N communication (see Figure 10a) and N2S communication (see Figure 10b). The results show that both N2N communication and N2S communication do not meet Scyther’s automatic declaration requirements of Alive, Weakagree (weak agree), Nisynch (noninjective synchronization), and Niagree (noninjective agreement). Moreover, the results also indicate that the secret session key *T_AB_* in the N2N communication and the secret session key *R_B_*_(*i*+1)_ in the N2S communication are both insecure. Hence, we conclude that the process of the authentication and key exchange in Roy et al.’s protocol is insecure and is subject to insider attacks on the IoT nodes and the server.

## 6. Suggestion for Preventing Insider Attacks

We know that *A* is required to recover *B*’s *R_Bi_* (see Equation (12)) to verify *B* (see Equation (14)) in the N2N communication of Roy et al.’s protocol. Similarly, *B* needs to recovering *A*’s *R_Ai_* (see Equation (19)) to verify *A* (see Equation (20)). However, we know that both *R_Ai_* and *R_Bi_* are the secrets of *A* and *B*, respectively. It means that *A* and *B* obtain each other secrets after Step 4 of the N2N communication, which incurs the secure breach. In fact, these verifications conducted by *A* and *B* are unnecessary because both *A* and *B* believe the server. Therefore, one suggestion is to cancel the verifications, that is, the server does not send *M_B_* and *H_B_* to *A* and *M_A_* and *H_A_* to *B* during Step 2 of the N2N communication.

Moreover, we find that both *A* and *B* share a random number *RN* in the N2N communication. *RN* is used to encrypt their all secrets. Because of sharing the *RN*, the malicious IoT node can decrypt other IoT node’s secrets. Hence, another suggestion is that the server must randomly select two random numbers instead of just one *RN*, that is, one random number for *A* and the other one for *B*.

The above two suggestions may defend against our proposed insider attacks. However, accurate security results for improvements require formal security models, security assumptions, security definitions, and reductions. We keep these for future work.

## 7. Conclusions

In the N2N communication of Roy et al.’s protocol, the IoT node requires the counterpart IoT node’s secret output of the PUF to verify it. More importantly, the malicious IoT node can derive the counterpart IoT node’s next output of the PUF if it eavesdrops on the message transmitted to the server. These defects have led to our insider attacks on Roy et al.’s protocol. Moreover, in the cases of the N2N communication and the N2S communication, we must point out that once the attacker stole the session keys, i.e., *T_AB_* or *R_B_*_(*i*+1)_, he can always impersonate not only the IoT node to cheat other IoT nodes or the server but also the server to cheat the IoT nodes in the subsequent sessions. The secure communications between the server and the corresponding IoT nodes are also possibly under the attacker’s surveillance. This means that Roy et al.’s protocol fails to provide the known key security. Our security analysis results indicate that designing PUF-based authentication and key exchange protocols for IoT remains a challenging task.

## Figures and Tables

**Table 1:** Used notation and symbols.

Notation	Description
*A*, *B*, *C*	IoT node *A*, IoT node *B*, IoT node *C*
*id_A_*, *id_B_*, *id_C_*	*A*’s identifier, *B*’s identifier, *C*’s identifier
*C_Ai_*, *C_Bi_*, *C_Ci_*	*A*’s *i*th iteration PUF input, *B*’s *i*th iteration PUF input, *C*’s *i*th iteration PUF input
*R_Ai_*, *R_Bi_*, *R_Ci_*	*A*’s PUF output for *C_Ai_*, *B*’s PUF output for *C_Bi_*, *C*’s PUF output for *C_Ci_*
*RN*	Random number
*T_AB_,T_CB_*	Secret sharing key between *A* and *B*, secret sharing key between *C* and *B*
*f^A^_PUF_*, *f^B^_PUF_*, *f^C^_PUF_*	*A*’s PUF function, *B*’s PUF function, *C*’s PUF function
⊕	Bit-wise XOR operation
*hash*()	Cryptographic hash function

**Table 2:** Scyther tool parameters used for our analysis.

Parameters	Parameter Specification
Max. number of runs	5
Matching type	Typed matching
Search pruning	Find best attack
Max. patterns/claim	10
Long-term key reveal	None
Long-term key reveal after claim	None(DY)
Session key reveal	Checked
Random reveal	Checked
State reveal	None

## Data Availability

No new data was created or analyzed in this study. Data sharing is not applicable to this article.
